# Hydrogen Permeability of Composite Pd–Au/Pd–Cu Membranes and Methods for Their Preparation

**DOI:** 10.3390/membranes13070649

**Published:** 2023-07-06

**Authors:** Polina Pushankina, Georgy Andreev, Iliya Petriev

**Affiliations:** 1 Department of Physics, Kuban State University, Krasnodar 350040, Russia; 2 Laboratory of Problems of Stable Isotope Spreading in Living Systems, Southern Scientific Centre of the RAS, Rostov-on-Don 344006, Russia

**Keywords:** membrane technologies, palladium-containing films, surface modification, nanostructured surface, pentagonal structured particles, catalytic activity, hydrogen permeability, hydrogen carriers, high-purity hydrogen

## Abstract

Thin Pd–40%Cu films were obtained via the classical melting and rolling method, magnetron sputtering, and modified with nanostructured functional coatings to intensify the process of hydrogen transportation. The films were modified by electrodeposition, according to the classical method of obtaining palladium black and “Pd–Au nanoflowers” with spherical and pentagonal particles, respectively. The experiment results demonstrated the highest catalytic activity (89.47 mA cm^−2^), good resistance to CO poisoning and long-term stability of Pd–40%Cu films with a pentagonal structured coating. The investigation of the developed membranes in the hydrogen transport processes in the temperature range of 25–300 °C also demonstrated high and stable fluxes of up to 475.28 mmol s^−1^ m^−2^ (deposited membranes) and 59.41 mmol s^−1^ m^−2^ (dense metal membranes), which were up to 1.5 higher, compared with membrane materials with classic niello. For all-metal modified membranes, the increase in flux was up to sevenfold, compared with a smooth membrane made of pure palladium, and for deposited films, this difference was manyfold. The membrane materials’ selectivity was also high, up to 4419. The developed strategy for modifying membrane materials with functional coatings of a fundamentally new complex geometry can shed new light on the development and fabrication of durable and highly selective palladium-based membranes for gas steam reformers.

## 1. Introduction

The rapidly developing hydrogen energy and high-tech industries are increasingly encouraging the development of hydrogen generation technologies. Currently, the most popular and effective technology for high-purity hydrogen evolution is membrane separation. Over the past few decades, membrane technology has been developed and investigated to meet various requirements of different applications [1,2,3,4,5,6,7]. It has a number of advantages, such as low energy consumption, the ability to conduct continuous separation, easy scaling, and the ability to combine with other separation technologies [8,9]. Palladium membranes are most preferred in industrial hydrogen separation conditions. [10,11,12]. This is due to the high ability of palladium to transport hydrogen in a wide temperature range, due to the higher solubility and diffusion ability of the FCC lattice. However, the practical application of membranes based on pure palladium is limited, since the metal tends to embrittlement in a hydrogen atmosphere [13,14]. Therefore, it is very difficult and sometimes even impossible to use the membranes for hydrogen separation, due to lattice expansion caused by dissolved hydrogen at temperatures below 300 °C [15,16]. This fact is due to the two immiscible Pd α- and β-phases, the first of which is a solid solution, and the second of which is palladium hydride [17,18]. There is a transition between the α- and β-phases in which the lattice space increases up to 10%. This leads to a lattice distortion, the formation of high internal stresses, deformation, and, ultimately, membrane destruction [19]. In addition to embrittlement, palladium is quite susceptible to poisoning and surface contamination by impurity gases, that contain sulfur or carbon compounds [20,21,22]. In addition, a significant difficulty in the fabrication of membranes based on pure palladium is the high metal cost [23,24]. Alloying palladium with other metals, such as silver, gold, copper, ruthenium, or nickel, makes it possible to overcome these difficulties [25,26,27,28]. For example, Q. Zhou et al. have studied gas separation properties of membranes based on Pd–Au and Pd–Au-Ag alloys [29]. Their results demonstrated the lowest hydrogen gas adsorption energy of −0.017 eV and −0.010 eV for Pd–Au and Pd–Au–Ag and excellent hydrogen selectivity and permeability characteristics of the developed membranes. Z. Han et al. [30] have investigated the characteristics of hydrogen separation by Pd-based alloy membranes using density functional theory (DFT) modeling and molecular dynamics calculations. According to their results, the Pd–Cu and Pd–Ni membranes showed excellent hydrogen selectivity compared with nitrogen, carbon monoxide, carbon dioxide, methane, and hydrogen sulfide at various temperatures, and the hydrogen permeability exceeded the limits of industrial production. S. Agnolin et al. [31] described the fabrication of thin Pd–Ag membranes on Hastelloy X tubular filters. A thin membrane layer was deposited on the surface of a tube modified by polishing and applying a smoothing interdiffusion barrier layer based on a boehmite dispersion. The filter pre-treatment was critical to increase the permeability and hydrogen selectivity of the final membrane.

One of the most promising membrane systems is the Pd–Cu binary alloy, which has good mechanical properties, high thermal stability, excellent hydrogen selectivity and permeability, relatively low cost, is resistant to hydrogen sulfide poisoning, and prevents hydrogen embrittlement at low temperatures [32,33,34]. According to the phase diagram [35,36], Pd–Cu alloys mainly have two different crystal lattices, face-centered cubic (fcc), and body-centered cubic (bcc) [37,38]. The most attractive structure for membrane alloys manufacture is the bcc structure, since such Pd–Cu alloys demonstrate the highest hydrogen permeability compared to those using a fcc structure [39,40,41]. Pd–40%Cu with a bcc structure and the highest hydrogen permeability seems to be the most promising of the variety of known alloys [42,43].

Another important factor in improving Pd-based membranes’ efficiency and durability is membrane film thinning. The deposition of a thin film on porous supports allows an increase in the hydrogen penetration rate and reduces the cost of the material, compared with the traditional Pd dense metal membranes [44,45,46]. Currently, the most common methods for manufacturing such a composite membrane are vacuum deposition, chemical vapor deposition, and chemical and galvanic deposition [47,48,49]. Among these methods, magnetron sputtering has a clear advantage, since it provides the synthesis of ultrathin films with minimum impurities, greater flexibility in the alloy synthesis, ease of control of process parameters, and the possibility of creating a nanostructured film [50,51].

However, another significant problem with hydrogen membrane separation currently is the extremely small and unstable or non-existent permeability of palladium-containing membranes at low temperatures (less than 200 °C). This fact is caused by the kinetic inhibition of establishing the equilibrium of metal–hydrogen systems, which is mainly due to an inactive or contaminated metal surface [52]. In this case, surface processes limit hydrogen transportation. The limit, which slows down the establishment of equilibrium between molecular hydrogen in the gas phase and atomic hydrogen absorbed in the palladium phase, can be partially overcome by increasing the surface roughness coefficient. “Catalytically active” cracks facilitate the establishment of equilibrium with hydrogen gas. They occur during alternating electrolytic oxidation and reduction, prolonged exposure to a glow discharge or calcination in air. Therefore, N. Vicinanza et al. [53] subjected Pd77%–Ag23% membranes to a three-stage heated air treatment to investigate the positive effects of such treatment on hydrogen transfer. It was found that air heat treatment affects the roughness and increases the effective membrane surface area, which increases the hydrogen permeability after each of the stages. In particular, surface activation can be carried out by applying a “hydrogen carriers” coating (modifier). There are various methods, such as the reduction in metal ions in solution, growth in the gas phase, evaporation on a substrate, and electrochemical deposition, which are promising for obtaining modifying coatings. “Hydrogen carriers” are hydrogen-chemisorbing substances such as platinum metals [54]. The nanostructured palladium layer formation on the membrane surface will increase the actual working surface area, thus leading to an increase in the number of chemisorption centers [55,56]. The deposition of a modifier based on pentagonal structured, multiply twinned nanoparticles is particularly interesting and effective. Membranes modified with such a coating make it possible to increase hydrogen permeability by several times in the low-temperature operating range [57,58]. The novelty of the work is the study of the effect of surface modifiers of various morphologies on membrane materials in low-temperature hydrogen transportation.

Accordingly, the aim of this work was to intensify the process of low-temperature hydrogen permeability of palladium–copper membranes by modifying the surface with a nanostructured coating composed of pentagonal, multiply twinned palladium–gold particles.

## 2. Materials and Methods

### 2.1. Methods for Creating and Studying Membrane Materials

Thin films of the Pd–40%Cu alloy were obtained by three methods:

The first method was to obtain a homogeneous Pd–Cu alloy by melting the constituent metal components of palladium and copper in an electric arc furnace in an inert argon atmosphere. Metals in the form of Pd ingots and oxygen-free copper were placed in a copper crucible for melting. The pressure inside the chamber was 0.05 MPa. During the melting process, the inverter current increased from 30 to 90 A. Subsequently, the obtained Pd–40%Cu ingot was rolled out on rollers to a film thickness of 20 μm with intermediate annealings.

The second method consisted in obtaining dense defect-free films of the Pd–Cu alloy with thickness up to 300 nm on a hydrogen-permeable aluminium oxide substrate by magnetron sputtering from a dense Pd–40%Cu target. Substrates with a thickness of 1.2 mm and an average pore size of 4 nm were purchased from the company «Germes technical components». The target was produced with the first method, i.e., by alloying components and rolling up to 40 µm with intermediate annealings. The films were deposited at a current of 40–50 mA on both sides of the substrate.

The third method for obtaining Pd–40%Cu on the surface of the substrate is the same as the previous one. However, the main difference between the third method and the second one is the usage of a composite target, which consists of palladium and copper plates with an area ratio of 60:40. The main advantage of this method is that the elements percentage in the alloy can be changed easily without the melting procedure and any changes in the resulting alloy structure.

The obtained films phase composition was determined on a Shimadzu XRD-7000 X-ray diffractometer (Shimadzu, Kyoto, Japan). The samples were studied in the range of 2θ angles from 30° to 80° with a scanning step of 0.02° using CuKα radiation (λ = 1.5406 Å) at a current of 30 mA and an accelerating voltage of 40 kV.

The chemical composition of the obtained alloys was controlled by microrentgenospectral analysis on an INCA (Oxford, UK) semiconductor energy dispersion attachment, which is a part of the JEOL JSM-7500F scanning electron microscope (JEOL, Tokyo, Japan).

### 2.2. Synthesis and Study of the Morphology of Nanostructured Coatings

The samples of the developed Pd–40%Cu alloy films were modified by two methods:

The first method of synthesis is the method of classical palladium black, which was carried out via electrochemical deposition from a palladium chloride solution using Elins P-40X potentiostat-galvanostat (Electrochemical Instruments, Chernogolovka, Russia). Palladium–copper films were prepared preliminarily by washing and degreasing; following this, samples were fixed in an electrolytic cell for polarization. Anodic polarization was carried out in HCl at a current density of 10 mA cm^−2^, and then cathodic polarization was carried out in H_2_SO_4_ at the same current. After pretreatment, the cell with prepared electrodes was filled with a palladium chloride solution. Deposition was performed at a current density of 6 mA cm^−2^ on both sides of the films.

The second method—Pd–Au nanoflowers—was based on the classical palladium black method; therefore, the synthesis was carried out according to a similar algorithm. A significant difference from the previous method was the synthesis process itself. The prepared electrodes were placed in a working cell filled with a growth solution of palladium chloride with a surfactant tetrabutylammonium bromide. Electrodeposition was carried out on a palladium–gold alloy electrode at a current density of 3 mA cm^−2^ on both sides of the films to obtain palladium–gold particles. All reagents were supplied by Sigma-Aldrich.

The samples’ surface morphology was investigated via the JEOL JSM-7500F scanning electron microscope (JEOL, Tokyo, Japan).

### 2.3. Study of Developed Materials in Catalytic and Membrane Applications

The catalytic activity of the modified films was studied with cyclic voltammetry (CV) in the reaction of alkaline methanol oxidation in the potential range from −0.9 to +0.5 V at a scanning rate of 50 mV s^−1^. The composition of the working solution was 1 M NaOH + 0.5 M methanol. The measurements were carried out on a P-40X potentiostat-galvanostat (Electrochemical Instruments, Chernogolovka, Russia) in a three-electrode cell. The working electrode was samples of modified Pd–40%Cu films, the counter electrode was a platinum electrode, and the reference electrode was a silver chloride glass electrode (Ag/AgCl), relative to which the potentials were applied.

The long-term stability of the developed nanoparticles as catalysts was studied with chronoamperometry in the reaction of alkaline methanol oxidation at a constant potential of −0.3 V during 0–2400 s.

The study of the hydrogen transfer processes through the developed samples of membrane materials was carried out with the special measuring hydrogen permeability device according to the method described in the work [59].

The selectivity of the developed membrane samples was studied through the ratio of passing flows of hydrogen and nitrogen (H_2_/N_2_). The nitrogen concentration in the hydrogen stream was determined using a flow chromatograph.

## 3. Results and Discussion

### 3.1. Structure and Characteristics of the Developed Membrane Materials

**Figure 1:**
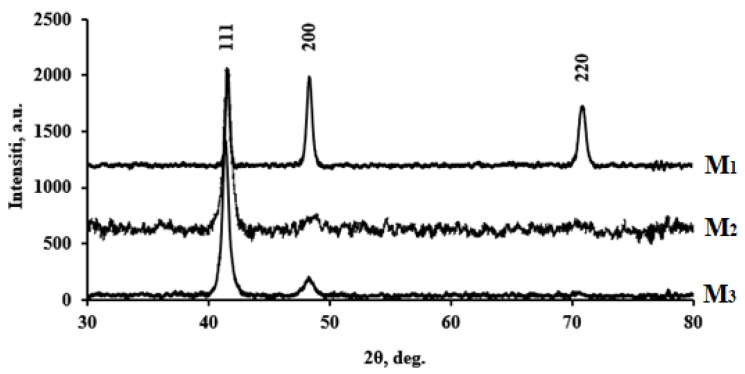
X-ray diffraction spectra of Pd–40%Cu alloy films.

Membrane materials obtained by three mentioned methods were characterized using the XRD method. X-ray diffraction spectra of Pd–40%Cu alloy films obtained by melting and classical rolling (M_1_), magnetron sputtering from a dense target (M_2_) and a composite target (M_3_) are shown in Figure 1. The face-centered cubic crystal structure (fcc) of palladium is characterized by four peaks at 2θ values equal to 40.1, 46.7, 68.2, 82.2 and corresponding to the planes (111), (200), (220) and (311), respectively. Four peaks at 2θ values equal to 43.3, 50.4, 74.1, and 89.9 characterized the crystal copper structures [60]. X-ray diffraction patterns of the samples shown in Figure 1 demonstrate appearance of each diffraction peak between the corresponding positions of the Pd and Cu fcc structure peaks, which confirms the successful formation of the Pd–Cu alloy.

It is known [61] that diffraction peaks are shifted to higher values of 2θ due to a lattice constant decrease, which is associated with a smaller diameter of copper atoms in comparison to palladium ones. For the examined samples 2θ values were 41.6, 48.3, and 70.9, corresponding to the (111), (200), and (220) planes, respectively. The lattice parameters (a) for bulk palladium and copper are 3.891 Å and 3.615 Å, respectively [62]. The lattice parameters for the studied samples and literary analogues are given in Table 1. The obtained data also confirm the formation of a single fcc structure. The sizes of crystallites (D) (regions of coherent scattering) were also calculated using the Scherrer formula (Table 1). Data analysis leads to a conclusion that the samples obtained by two methods of magnetron sputtering are characterized by a smaller crystallite size, compared with the samples obtained by alloying and rolling.

**Figure 2:**
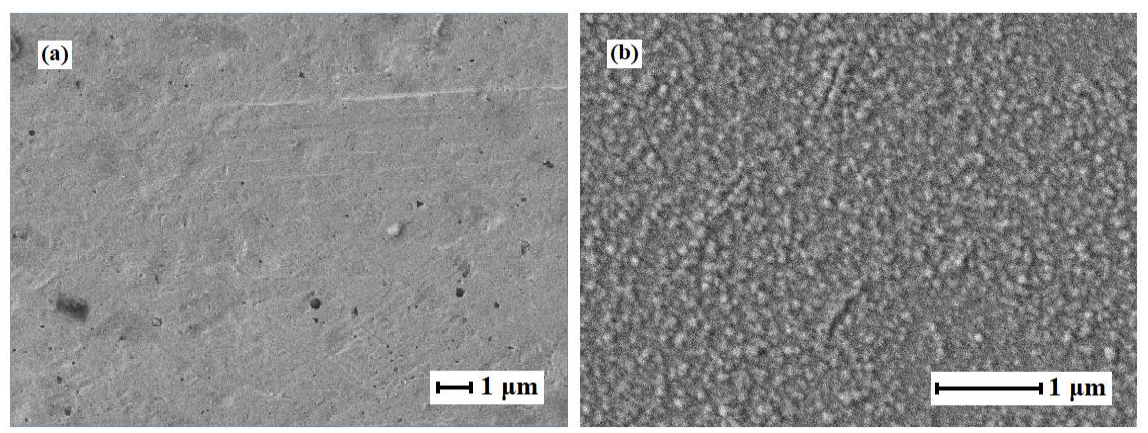
SEM images of the Pd–40%Cu membrane obtained by alloying components and rolling (**a**) and by magnetron sputtering (**b**).

Pd–40%Cu films obtained by alloying and rolling methods had a relatively homogeneous surface. SEM images of the Pd–40%Cu membrane surfaces studied in the work are shown in Figure 2.

### 3.2. Morphology of Nanostructured Coatings and Catalytic Characteristics of Modified Films

**Figure 3:**
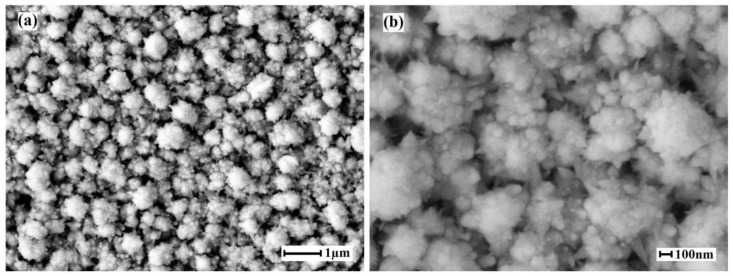
SEM images of the surface of Pd–40%Cu films modified by the classical palladium black method at magnifications of 10,000 (**a**) and 30,000 (**b**).

The nanostructured coating on the Pd–40%Cu alloy film surfaces synthesized by the classical palladium black method consisted of typical spherical particles with characteristic sizes in the range of 90–120 nm. SEM images of the films surface modified by the classical palladium black method, are shown in Figure 3. After applying the modifier on the Pd–40%Cu films surface, the real working surface area increased up to 9.4 times and the coating roughness coefficient increased to 15.13.

**Figure 4:**
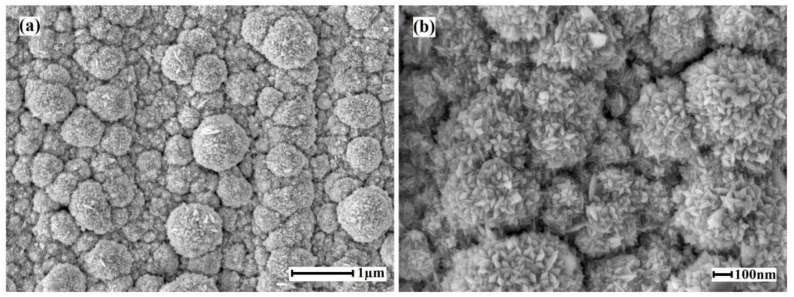
SEM images of the surface of Pd–40%Cu films modified by the “Pd-Au nanoflowers” method at magnifications of 10,000 (**a**) and 30,000 (**b**).

The nanostructured coating on the of Pd–40%Cu alloy films surfaces synthesized by the “nanoflowers” method consisted of pentagonal structured Pd-Au nanoparticles with multiple corrugations with characteristic sizes in the range of 100–130 nm. To obtain such coatings with a large number of active centers, the particles growth was directed towards high-index facets. SEM images of the surface of the films modified with the “Pd–Au nanoflowers” method are shown in Figure 4. After applying the modifier on the Pd–40%Cu films’ surface, the real working surface area increased up to 11.2 times and the coating roughness coefficient increased to 18.02. The results of the EDS analysis showed 95.12% palladium and 4.88 % gold content in the synthesized functional layer.

**Figure 5:**
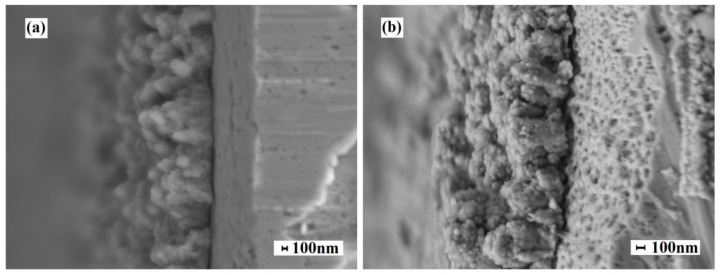
SEM images of cross-sections of modified by the “Pd–Au nanoflowers” method samples of Pd–40%Cu films obtained by alloying and rolling (**a**) and magnetron sputtering (**b**).

The average thickness of the modifying layer was about 0.7 μm. SEM images of cross-sections of modified Pd–40%Cu films samples obtained by alloying and rolling method and magnetron sputtering method are shown in Figure 5.

**Figure 6:**
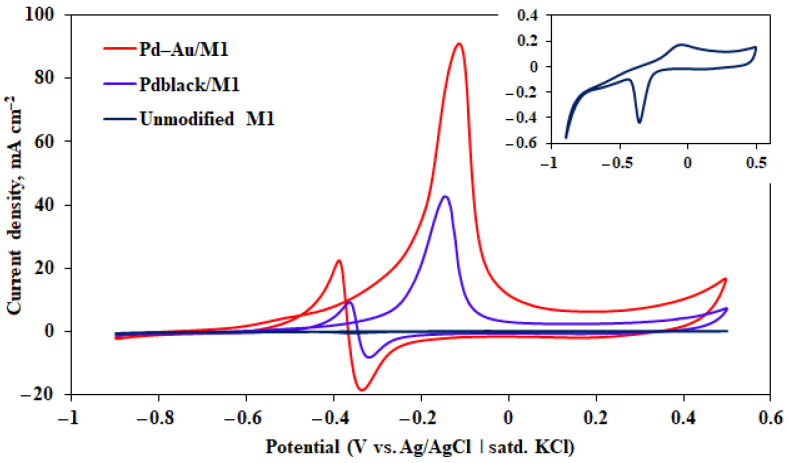
Established CV of Pd–40%Cu films.

The developed Pd–40%Cu M_1_ film samples, modified with two types of coatings, namely pentagonally structured coating (Pd–Au) and classic black one (Pd_black_), were studied in the alkaline methanol oxidation reactions. Two peaks can be observed on all current-voltage curves in the potential range from −1 to 0.5 V, which can be seen in Figure 6. The oxidation peak in the forward scan is related to the chemisorbed methanol electrooxidation, and the oxidation peak in the reverse one is associated with the removal of carbonaceous intermediates that were not completely oxidized in the forward scan. The data demonstrate a significant increase up to 89.47 mA cm^−2^ in the peak current density of Pd–Au/M_1_ samples. These peak current density values are up to 2 times higher than the ones for Pd_black_/M_1_ films (up to 40.38 mA cm^−2^), and up to several dozen times than unmodified M_1_ film values (up to 0.17 mA cm^−2^). The peak potential shift is another important activity characteristic of the electrodes in relation to the methanol oxidation reaction. With a further increase in the potential, the current density begins to decline due to an increase in the coverage of CO_ads_ on the active centers and depletion of methanol near to the electrode surface. It should also be noted that the synergistic effect of bimetallic compositions of palladium with gold plays one of the key roles in the catalytic activity increase. This combination makes it possible to inhibit the poisoning of palladium active sites most effectively due to the ability of gold to accelerate the oxidation of intermediate reaction products [64].

The catalysts resistance to CO poisoning was evaluated in terms of the ratio of the current peaks appearing during the forward and reverse scans [65]. High values of the ratio indicate effective desorption of CO on the developed modified electrodes, and on the contrary, low values indicate an excessive accumulation of residual CO_ads_ forms on the surface. The highest stability index was demonstrated by Pd–Au/M_1_ samples—4.5. Pd_black_/M_1_ samples also showed good stability—4.

**Figure 7:**
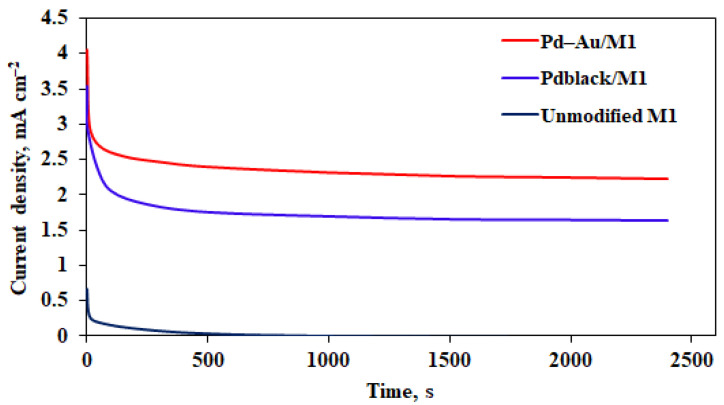
CA curves of methanol oxidation of Pd–40%Cu films.

Another critical aim in the design and development of efficient catalyst systems is to solve stability problems caused by rapid carbonaceous intermediates absorption [66]. The catalysts durability and electrochemical stability were studied with chronoamperometry (CA) at a constant oxidation potential of −0.3 V. Figure 7 shows the measured durability curves of Pd–Au/M_1_ and Pd_black_/M_1_ samples. It can be seen in Figure 7 that a sharp current decrease was fixed in the first seconds of measurements for all samples, before its stable value was reached. This observation is typical and is associated with the continuous oxidation of methanol at the maximum potential, the formation of a certain amount of PdO and residual COads particles on the surface of the samples during the electrooxidation of methanol [67]. The current decrease in Pd_black_/M_1_ samples is more noticeable. It is associated with the adsorption of intermediates formed during methanol oxidation reaction. It can be seen from the CA analysis that the Pd–Au/M_1_ sample has a stable higher stationary current density in the methanol oxidation reaction up 2.23 mA cm^−2^, compared with the Pd_black_/M_1_ (1.63 mA cm^−2^) and unmodified M_1_ (0.01 mA cm^−2^) samples. This result may be due to a small amount or complete absence of adsorbed intermediates on the catalyst surface. Probably, the main reason for the observed higher stability of Pd–Au/M_1_ samples is the presence of a large number of particles with high-index facets on the surface of Pd–40%Cu films, which lead to the rapid oxidation of adsorbed intermediates with the formation of a large number of active centers. The increased stability of the Pd–Au/M_1_ samples is also due to the inclusion of gold, which reduces the CO of electrode surface and increases the electronic conductivity [68]. The current decrease is much faster due to the possibility of greater carbon adsorption during the methanol oxidation reaction in the case of Pd_black_/M_1_ samples with pure palladium.

The study of the developed modifying coatings deposited on Pd–40%Cu films in the methanol oxidation reaction demonstrated a significant increase in the catalytic activity, resistance to CO and stability of Pd–Au/M_1_ films in comparison Pd_black_/M_1_. The result for the modified films is probably due to an increased number of localized areas of high-energy surface centers, and a synergistic effect from the secondary metal (gold) in the composition of the nanoparticles. The formation of active centers in the coating synthesized by the “Pd–Au nanoflowers” method occurs due to multiple twinning of nanoparticles with the high-index facets with a large number of hydrogen-reactive insufficiently coordinated atoms.

### 3.3. Investigation of Modified Membrane Materials in Hydrogen Transport Processes

**Figure 8:**
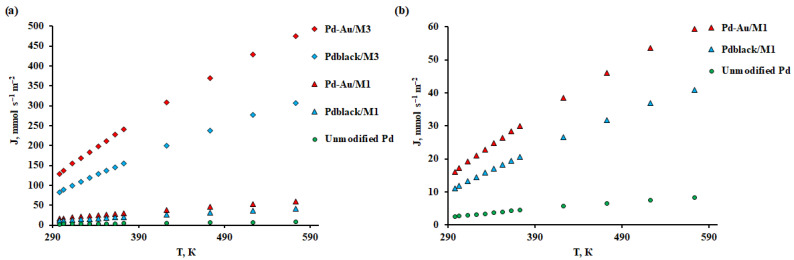
(**a**) Temperature dependence of the hydrogen flux at gauge pressure of 0.1 MPa through modified Pd–40%Cu membranes and an unmodified pure palladium membrane. (**b**) Temperature dependence of the hydrogen flux at gauge pressure of 0.1 MPa through modified dense-metal Pd–40%Cu membranes and an unmodified pure palladium membrane.

The synthesized nanoparticles were studied as modifying coatings in hydrogen transport processes. The membrane substrates were Pd–40%Cu M_1_ and M_3_ films. The measurements were carried out in the temperature range from 25 to 300 °C, because the effect of the applied modifiers is expected exactly in this range. According to the data presented in Figure 8, Pd–Au/M_1_ and Pd-Au/M_3_ membranes had the highest flux values up to 59.41 mmol s^−1^ m^−2^ and 475.28 mmol s^−1^ m^−2^ at 300 °C, respectively. The Pd_black_/M_1_ and Pd_black_/M_3_ membranes showed a flux 1.5 times less than the previous ones, up to 40.97 mmol s^−1^ m^−2^ and 307.29 mmol s^−1^ m^−2^, respectively. The density values of penetrating fluxes of deposited and dense-metal Pd–40%Cu films obtained by one method and modified by different types of coatings were in the same range. The difference in the ranges of films obtained by different methods was about 8 times. Such a difference in the flux density is due to the difference in the thickness of the obtained films. This effect can be explained by a significant leveling of the contribution of surface processes to the limitation of hydrogen transfer through palladium-containing membranes.

The results achieved in this experiment became possible due to the limitation of hydrogen transportation by dissociative-associative processes on the surface, since the state of the surface has a significant effect in this temperature range (25–300 °C). The application of a nanostructured modifier to the membrane sample surfaces leads to both an increase in the roughness of the membrane surface as well as an increase in the active centers number. This is especially noticeable for Pd–Au/M_1_ and Pd–Au/M_3_ membranes. The high concentration of such catalytic sites is probably due to the growth features for a given geometric shape of nanoparticles.

**Figure 9:**
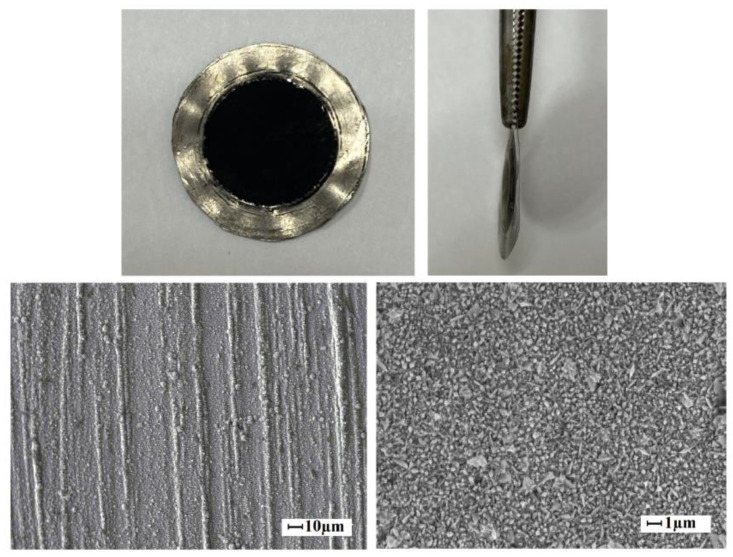
Photo and SEM image of the membrane surface after experiments in a hydrogen atmosphere at gauge pressure of 0.5 MPa in the temperature range of 25–300 °C.

The membranes retained their integrity, and the modifying coatings also retained their mechanical integrity without visible changes in the surface condition after experiments in a hydrogen atmosphere. Photos and SEM image of the membrane after experiments in a hydrogen atmosphere are shown in Figure 9.

**Figure 10:**
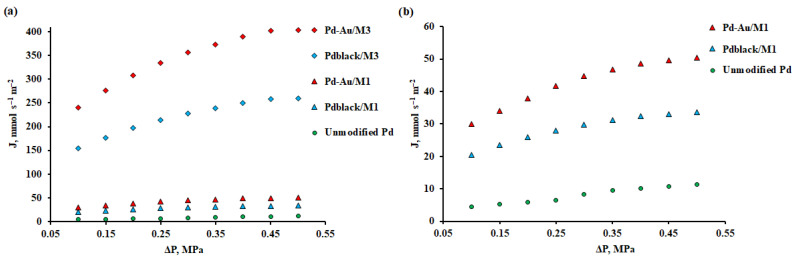
(**a**) Dependence of the hydrogen flux on the inlet side gauge pressure at 100 °C of modified Pd–40%Cu membranes and an unmodified pure palladium membrane. (**b**) Dependence of the hydrogen flux on the inlet side gauge pressure at 100 °C of modified dense-metal Pd–40%Cu membranes and an unmodified pure palladium membrane.

The films’ permeability was also measured depending on the pressure change in the range from 0.1 to 0.5 MPa to determine the limiting stage of hydrogen transportation. The experiment was carried out at a temperature of 100 °C, which is of the greatest interest, since it makes it possible to observe the effect of surface processes on permeability most clearly. As can be seen in Figure 10, the data obtained for an unmodified pure palladium membrane are well approximated by a first-order curve, and the exponent n is close to 1. This result indicates that surface effects limit the process of hydrogen transfer through a smooth palladium membrane completely. In the case of Pd_black_/M_1_ and Pd_black_/M_3_ membranes, the exponent *n* has the value up to 0.9 and 0.89, respectively, which indicates that the hydrogen transport process is limited by the combination of surface stages and diffusion, but the role of the first ones is still more prevalent. The Pd–Au/M_1_ and Pd–Au/M_3_ membranes show a more parabolic curve with an *n* up to 0.75 и 0.74, respectively. This value is close to 0.5, which indicates a partial removal of surface limits and a greater transition to a diffusion-limited regime.

**Figure 11:**
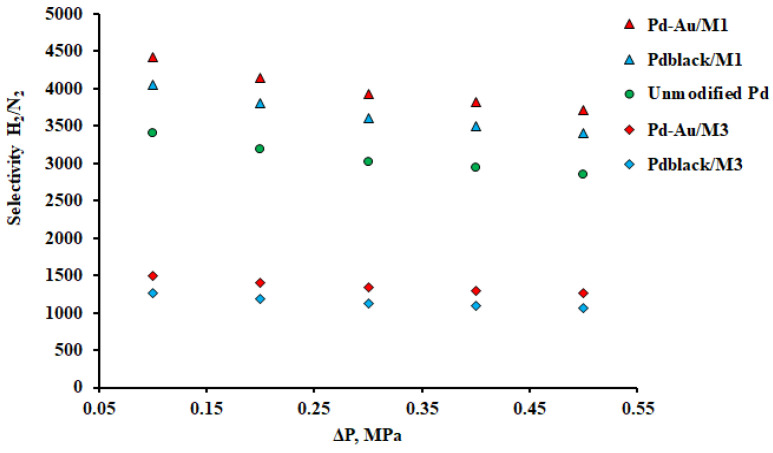
Dependence of H_2_/N_2_ selectivity on gauge pressure at the inlet side of Pd–40%Cu membranes and an unmodified pure palladium membrane.

The selectivity of the developed modified membranes was studied through the ratio of passing H_2_ to N_2_ flows under conditions of a pressure drop in the retentate zone from 0.1 to 0.5 MPa. According to Figure 11, all investigated membranes showed a high level of selectivity. Pd–Au/M_1_ and Pd_black_/M_1_ films had the highest selectivity up to 4419 at 0.5 MPa. The selectivity of the Pd–Au/M_3_ and Pd_black_/M_3_ films was 3.5 times lower than the selectivity of the Pd–Au/M_1_ and Pd_black_/M_1_ films. Lower selectivity values of Pd–Au/M_3_ and Pd_black_/M_3_ membrane samples indicate the presence of minor defects or a less dense crystal lattice. It should be noted that the selectivity was quite close for films obtained by the same method, but modified with different coatings. For Pd–Au/M_1_ and Pd_black_/M_1_ films the difference was 1.1 times (4419 and 4055, respectively). For Pd–Au/M_3_ and Pd_black_/M_3_ films the difference was 1.2 times (1503 and 1263, respectively). The experiment was carried out with an increase in pressure up to 0.5 MPa and a further decrease to a starting value of 0.1 MPa. At the end of the experiment, the hysteresis dependence was not registered, which confirms the ability of the developed membranes to withstand pressure drops. As the pressure on the inlet side of the membranes increased, a decrease in selectivity was observed, which can be seen in Figure 11. Nevertheless, the numerical selectivity decrease can be considered insignificant. The results allow to assume that there are no significant defects in the developed films and the studied membrane samples are stable against pressure drops.

A comparative analysis of the result obtained in this work with the available literature data was carried out to evaluate the efficiency of the developed Pd–Cu membranes (Table 2). However, there are quite a few works with stable and reproducible experimental data on the low-temperature permeability of hydrogen through the palladium phase. Moreover, the existing literature reports on obtaining data vary greatly in the quantitative expression of the obtained values. There are practically no works demonstrating the results of experiments on the hydrogen permeability of palladium membranes below 100 °C.

## 4. Conclusions

In this study, a high hydrogen yield was achieved by using thin Pd–40%Cu membranes modified with a pentagonal structured Pd–Au coating. Self-supporting Pd–40%Cu films obtained by melting and rolling and films obtained by magnetron sputtering from a Pd–40%Cu target on a substrate were modified in order to intensify low-temperature hydrogen transportation. Classical palladium black and pentagonal structured Pd–Au particles, obtained with method of controlled synthesis, were used as surface modifiers. The integration of the developed metal films and surface modifiers made it possible to achieve high catalytic activity and stability in the methanol oxidation reaction. The highest peak current densities were observed up to 89.47 mA cm^−2^ for Pd–Au/M_1_ films. The obtained values were 2.2 times higher than those of Pd_black_/M_1_ films (40.38 mA cm^−2^) and several dozen times higher than values of unmodified Pd–40%Cu film. Also, Pd–Au/M_1_ films had the best resistance to CO poisoning and higher stability (2.23 mA cm^−2^). This result may be due to both an increase in the number of localized areas of high-energy surface centers, and the presence of a synergistic effect from the secondary metal–gold in the composition of nanoparticles. The developed membrane materials were studied in the hydrogen transport processes in the temperature range from 25 to 300 °C. A significant hydrogen flux increase was experimentally recorded up to 475.28 mmol s^−1^ m^−2^ and 59.41 mmol s^−1^ m^−2^ at 300 °C for Pd–40%Cu Pd–Au/M_3_ and Pd–Au/M_1_ membranes respectively. Numerically, the increase was 1.5 times in comparison to samples of Pd_black_/M_3_ and Pd_black_/M_1_ membranes. For dense-metal-modified membranes, the increase in flux was up to 7 times, in comparison to a smooth membrane made of pure palladium, and for deposited films, this difference was dozens of times. The achievement of this result is due to an increase in the membrane surface roughness, as well as an increase in the number of active centers on the surface for modifiers with complex geometry, surface modification has a significant effect, since the hydrogen transfer process is limited by dissociative-associative stages in the selected temperature range. The experiments confirm that the deposition of a pentagonal structured Pd–Au coating on the surface of the developed Pd–40%Cu membranes can significantly reduce surface restrictions by a transition to a diffusion-limited mode. The H_2_/N_2_ selectivity for the developed modified membrane materials also showed high values and the absence of significant defects in the membranes. The strategy of modifying membrane materials with functional coatings of a fundamentally new complex geometry can shed new light on the development and production of durable and highly selective palladium-based membranes for gas steam reformers.

## Figures and Tables

**Table 1:** Parameters of the studied Pd–40%Cu films and literary analogues.

Film	Elemental Composition	a, Å	D, nm	Reference
Pd–Cu M_1_	Pd 60.08 Cu 39.92	3.76 ± 0.002	30.5	This work
Pd–Cu M_2_	Pd 59.96 Cu 40.04	3.76 ± 0.06	21.1	This work
Pd–Cu M_3_	Pd 60.31 Cu 39.69	3.77 ± 0.003	22.1	This work
Pd–Cu	Pd 50 Cu 50	3.77	–	[11]
Pd–Cu	Pd 55.31 Cu 44.69	3.867	–	[17]
Pd–Cu	Pd 53.1 ± 0.4 Cu 46.9 ± 0.4	3.775	–	[36]
Pd–Cu	Pd 51.9 ± 0.4 Cu 48.1 ± 0.4	3.763	–	[38]
Pd–Cu	Pd 46 Cu 54	3.782	–	[47]
Pd–Cu	Pd 59.2 ± 0.8 Cu 40.8 ± 0.8	3.757 ± 0.003	–	[63]

**Table 2:** Hydrogen permeability and H_2_/N_2_ selectivity for the developed membranes and literature analogs.

Membrane	Support	Thickness, μm	J, mmol s^−1^ m^−2^	Temperature, K	∆p, kPa	Selectivity H_2_/N_2_	Reference
Pd	YSZ–Al_2_O_3_	5	450	623	390	350	[69]
Pd	Al_2_O_3_	10	95	573	200	176	[70]
Pd_75_Ag_25_	–	25	≈300	873	500	–	[71]
Pd_88_Ag_12_	Al_2_O_3_	11	200	623	160	2073	[72]
PdCu/Ta	–	250	5.2	673	100	–	[11]
50 vol. % Pd–GDC	–	282	41	1173	<100	–	[73]
50 vol. % Pd–CZY	–	500	17	1173	<100	–	[74]
50 vol. % Pd–YSZ	–	218	24	1173	<100	–	[75]
Pd_70_Cu_30_	Al_2_O_3_	20	105	573	100	1500	[76]
Pd_66_Cu_34_	Al_2_O_3_	4	190	783	350	5000	[77]
Pd_47_Cu_53_	Al_2_O_3_–ZrO_2_	3.5	220	573	500	100	[33]
Pd	–	20	0.11	373	500	3399	This work
Pd–Au/M_1_	–	20	59.41	573	100	4419	This work
Pd_black_/M_1_	–	20	40.97	573	100	4055	This work
Pd–Au/M_3_	Al_2_O_3_	0.3	475.28	573	100	1503	This work
Pd_black_/M_3_	Al_2_O_3_	0.3	307.29	573	100	1263	This work

## Data Availability

Not applicable.
